# Resistance Development of Cystic Fibrosis Respiratory Pathogens When Exposed to Fosfomycin and Tobramycin Alone and in Combination under Aerobic and Anaerobic Conditions

**DOI:** 10.1371/journal.pone.0069763

**Published:** 2013-07-25

**Authors:** Gerard McCaughey, Paul Diamond, J. Stuart Elborn, Matt McKevitt, Michael M. Tunney

**Affiliations:** 1 CF and Airways Microbiology Research Group, Queen's University Belfast, Belfast, United Kingdom; 2 School of Pharmacy, Queen's University Belfast, Belfast, United Kingdom; 3 Centre for Infection and Immunity, School of Medicine, Dentistry and Biomedical Sciences, Queen's University Belfast, Belfast, United Kingdom; 4 Gilead Sciences, Inc., Seattle, Washington, United States of America; University of Tübingen, Germany

## Abstract

Although antibiotics from different classes are frequently prescribed in combination to prevent the development of resistance amongst Cystic Fibrosis (CF) respiratory pathogens, there is a lack of data as to the efficacy of this approach. We have previously shown that a 4∶1 (w/w) combination of fosfomycin and tobramycin (F∶T) has excellent activity against CF pathogens with increased activity under physiologically relevant anaerobic conditions. Therefore, the aim of this study was to determine whether F∶T could delay or prevent the onset of resistance compared to either fosfomycin or tobramycin alone under aerobic and anaerobic conditions. The frequency of spontaneous mutants arising following exposure to fosfomycin, tobramycin and F∶T was determined for clinical *Pseudomonas aeruginosa* and MRSA isolates under aerobic and anaerobic conditions. The effect of sub-inhibitory concentrations of fosfomycin, tobramycin and F∶T on the induction of resistance was also investigated, with the stability of resistance and fitness cost associated with resistance assessed if it developed. *P. aeruginosa* and MRSA isolates had a lower frequency of spontaneous mutants to F∶T compared to fosfomycin and tobramycin under both aerobic and anaerobic conditions. There was a maximum two-fold increase in F∶T MICs when *P. aeruginosa* and MRSA isolates were passaged in sub-inhibitory F∶T for 12 days. In contrast, sequential resistance to fosfomycin and tobramycin developed quickly (n = 3 days for both) after passage in sub-inhibitory concentrations. Once developed, both fosfomycin and tobramycin resistance was stable and not associated with a biological fitness cost to either *P. aeruginosa* or MRSA isolates. The results of this study suggest that F∶T may prevent the development of resistance compared to fosfomycin or tobramycin alone under aerobic and physiologically relevant anaerobic conditions. F∶T may be a potential treatment option in CF patients chronically colonised by MRSA and/or *P. aeruginosa*.

## Introduction

Resistance to antimicrobial agents is currently one of the major problems in the healthcare setting worldwide, with changes from susceptible to more resistant bacterial populations occurring as an evolutionary response to antibiotic usage [1]. Antibiotic resistance is particularly problematic in the treatment of chronic respiratory infection in Cystic Fibrosis (CF), the major cause of morbidity and mortality in this patient population [2]. Antimicrobial resistance is potentiated in CF patients due to the extensive use of antimicrobial agents from a young age, both for the prophylaxis and treatment of respiratory infection. The net result of this high antimicrobial usage is the sequential development of resistance to antibiotics amongst CF pathogens [3], with approximately 25–45% of CF patients now colonised with multidrug resistant (MDR) bacteria [4], [5]. It is also now widely recognised that the CF lung contains anaerobic regions, with these regions supporting the growth of obligate and facultative anaerobes [6]–[9]. Anaerobiosis can reduce the activity of some classes of antimicrobials such as the aminoglycosides leading to further problems with appropriate antimicrobial treatment selection [10], [11].

One potential strategy to prevent the development of resistance and to improve treatment outcome is to use a combination of two or more antimicrobials from different classes, especially if these antibiotics have a different mechanism of action. Antibiotics are routinely used in combination to treat CF lung infection [12]; however, data on the ability of these combinations to prevent the development of resistance is limited and the effect of anaerobic conditions on the development of resistance amongst CF pathogens has not been determined.

We have previously shown that a 4∶1 combination (w/w) of fosfomycin and tobramycin (F∶T) has excellent *in vitro* activity against MRSA and *P. aeruginosa* and importantly also has increased activity under physiologically relevant anaerobic conditions [13]. The aim of this study was to determine if the combination of fosfomycin and tobramycin in F∶T could prevent the onset of spontaneous and induced resistance in *P. aeruginosa* and MRSA isolates from CF patients under aerobic and anaerobic conditions. In addition, we aimed to determine if the development of fosfomycin, tobramycin or F∶T resistance was associated with a biological fitness cost in *P. aeruginosa* and MRSA isolates.

## Methods

### Bacterial isolates

Five clinical *P. aeruginosa* and five clinical MRSA isolates were used in this study. *P. aeruginosa* isolates were cultured from sputum samples collected from adult CF patients at the CF centre (Belfast, UK). MRSA isolates were cultured from bronchoalveolar lavage fluid collected from paediatric patients attending the University of North Carolina CF Centre (Chapel Hill, USA). In addition, *P. aeruginosa* strain ATCC 27853 and *S. aureus* strain ATCC 29213 were used as controls for susceptibility testing and were also used in spontaneous mutation frequency studies. Susceptibility (Minimum Inhibitory Concentration, MIC) of these strains as determined previously [13] is shown in Table 1.

**Table 1:** Fosfomycin, tobramycin and F∶T MICs for MRSA and *P. aeruginosa* strains used in this study.

Isolate	Species	Aerobic MIC (mg/L)			Anaerobic MIC (mg/L)		
		Fof	Tob	F∶T	Fof	Tob	F∶T
CF35	*P. aeruginosa*	8	4	20	16	16	20
P3	*P. aeruginosa*	16	4	10	8	16	20
CA6	*P. aeruginosa*	256	16	40	8	32	10
W050	*P. aeruginosa*	4	2	10	4	16	10
AY4	*P. aeruginosa*	8	4	10	16	16	2.5
27853	*P. aeruginosa*	4	1	5	4	4	5
25A	MRSA	8	0.5	5	1	2	5
CFP8	MRSA	2	0.5	5	1	4	2.5
CFP13	MRSA	1	1	5	1	4	2.5
M6	MRSA	8	0.5	2.5	2	4	1.25
M10	MRSA	8	1	2.5	4	8	5
29213	MRSA	4	0.5	2.5	0.5	32	2.5

### Antibiotics and reagents

Fosfomycin disodium was purchased from Ecros (Barcelona, Spain) and tobramycin sulphate was purchased from Chonqing Imperial Biochem (Chongqing, China) with both antibiotics United States Pharmacopeia (USP) grade. F∶T consisted of a 4∶1 (w/w) ratio of fosfomycin and tobramycin, with F∶T concentration expressed as a combination of both antibiotics (e.g.20 mg/L F∶T  = 16 mg/L fosfomycin +4 mg/L tobramycin). Mueller Hinton agar (MHA, Oxoid Ltd, Basingstoke, UK ) and Mueller Hinton broth (MHB; Oxoid Ltd, Basingstoke, UK) were supplemented with 1% (w/v) potassium nitrate (KNO_3_, Sigma-Aldrich, Gillingham, UK). when testing *P. aeruginosa* to act as the terminal electron receptor and allow for anaerobic respiration. Glucose-6-Phosphate (G6P, Sigma-Aldrich, Gillingham, UK) was added to the media at a final concentration of 25 mg/L for all experiments. Anaerobic conditions were achieved using an anaerobic workstation (Whitley A35 Anaerobic workstation, Don Whitley Scientific, Shipley, UK).

### Minimum Inhibitory Concentration (MIC) testing

MIC testing was carried out using the agar dilution method, as described previously [13], with doubling dilutions in the following antibiotic concentration ranges used: tobramycin, 0.125–32 mg/L; fosfomycin, 0.5–256 mg/L and F∶T, 0.625–320 mg/L. Isolates were then classified as sensitive, intermediate or resistant to fosfomycin and tobramycin according to the Committee for Laboratory Standards Institute (CLSI) breakpoints (Fosfomycin; ≤64 mg/L sensitive, 128 mg/L intermediate, ≥256 mg/L resistant. Tobramycin; ≤4 mg/L sensitive, 8 mg/L intermediate, ≥16 mg/L resistant) [14]. As no breakpoints are reported by the CLSI for *P. aeruginosa* or MRSA with fosfomycin, those provided for Enterobacteriaceae were used.

### Spontaneous mutation frequency

The frequency of spontaneous mutants arising with increased fosfomycin, tobramycin and F∶T MICs was determined for five clinical and one reference *P. aeruginosa* isolate and for five clinical MRSA and one reference *Staphylococcus aureus* isolate using a method modified from that described by MacLeod *et al* [15]. Briefly, isolates were inoculated in duplicate into 10 mls MHB with one aliquot incubated under aerobic conditions and one aliquot incubated under anaerobic conditions at 37°C. After growth to late log phase (10^8^–10^9^ cfu/ml), 100 µls of each culture was spread onto the surface of MHA plates containing fosfomycin, tobramycin and F∶T at 2×, 4× and 8× the MIC for each isolate. In addition, appropriate dilutions of each culture were plated onto antibiotic free MHA plates to determine the number of viable cells in the initial inoculum. After incubation at 37°C for 48 hours, the frequency of spontaneous mutants was calculated by dividing the number of colonies growing on the antibiotic containing plates by the number of viable cells. Fosfomycin, tobramycin and F∶T MICs of representative mutant isolates (n = 9) selected on fosfomycin and tobramycin plates were determined as above and compared to that of the parent strain.

### Induction of resistance

Development of resistance after serial exposure to fosfomycin, tobramycin and F∶T was determined for 3 clinical *P. aeruginosa* (AY4, W050, CF35) and 3 clinical MRSA (CFP8, M6, 25A) isolates. Isolates were inoculated into 10 mls MHB in duplicate, with one aliquot incubated under aerobic conditions and one aliquot incubated under anaerobic conditions at 37°C. The Optical Density (OD_550nm_) of the bacterial culture was adjusted to 0.15 (approx 1×10^8^ cfu/ml) after overnight growth and 50 µl was added to MHB containing sub-inhibitory concentrations of fosfomycin, tobramycin and F∶T (determined by growth curves, see Table S1 for concentrations) and control (no antibiotics added). The cultures were incubated overnight and this process was repeated daily for 12 days, standardising the inoculum to an OD_550nm_ of 0.15 and using the same antibiotic concentrations for each passage. Susceptibility testing was carried out after every 3 passages using the agar dilution method as described above, with the culture adjusted to OD_550nm_ 0.15 used as the initial inoculum. After 12 days, those isolates which had developed resistance to any of the antibiotics tested were passaged in antibiotic free broth for a further 12 days with MICs tested after every 3 passages. For each isolate, the MICs of the control were tested at days 1 and 12 to ensure passage alone had no effect on MIC.

### Fitness Cost

The fitness cost associated with the development of fosfomycin or tobramycin resistance was determined by comparing the growth of the resistant isolate with that of the susceptible parent isolate using growth curves. Overnight cultures were adjusted to an OD_550nm_ of 0.15 in MHB, further diluted 1∶10 and 50 µl added to a total volume of 20 mls MHB. Bacterial cultures were then incubated under aerobic and anaerobic conditions and total viable counts determined after 0, 1, 2, 4, 6 and 24 hours.

## Results

### Frequency of spontaneous *P. aeruginosa* and MRSA mutants with elevated MICs is lower with F∶T than fosfomycin or tobramycin alone

For *P. aeruginosa* isolates (n = 6), the frequency of spontaneous mutants was lower following exposure to F∶T compared to fosfomycin and tobramycin alone under both aerobic and anaerobic conditions as summarised in Table 2 (see Table S2 for full results). The frequency of mutants on exposure to F∶T was below the detectable limit in 31/36 (86%) experiments performed (n = 6 isolates at 2×,4× and 8× MIC under both aerobic and anaerobic conditions). In contrast, the frequency of *P. aeruginosa* mutants to fosfomycin and tobramycin was below the limit of detection in only 3/36 (8%) and 15/36 (42%) experiments, respectively. *P. aeruginosa* isolates exhibited lower frequency of mutants to fosfomycin under anaerobic (average 4.61×10^−6^ at 8× MIC) compared to aerobic conditions (average 2.24×10^−5^ at 8× MIC) whereas, frequency of mutants to F∶T and tobramycin were similar under both conditions.

**Table 2:** Mean^a^ (S.D.) frequency of spontaneous MRSA (n = 6) and *P. aeruginosa* (n = 6) mutants with increased fosfomycin (FOF), tobramycin (TOB) and F∶T MICs under aerobic and anaerobic conditions at 2×, 4× and 8× MIC.

Drug	Aerobic			Anaerobic		
	2× MIC	4× MIC	8× MIC	2× MIC	4× MIC	8× MIC
***P. aeruginosa***						
FOF	6.62×10^−5^ (8.7×10^−5^)	2.79×10^−5^ (4.6×10^−5^)	2.24×10^−5^ (4.8×10^−5^)	1.23×10^−5^ (1.2×10^−5^)	6.86×10^−6^ (9.7×10^−6^)	4.61×10^−6^ (1×10^−5^)
TOB	6.77×10^−6^ (1.3×10^−5^)	7.83×10^−6^ (1.5×10^−5^)	3.7×10^−6^ (8.9×10^−6^)	4.33×10^−5^ (9.1×10^−5^)	4.83×10^−6^ (4.5×10^−6^)	3.07×10^−6^ (4.6×10^−6^)
F∶T	5.55×10^−8^ (4.8×10^−8^)	5.55×10^−8^ (4.8×10^−8^)	5.55×10^−8^ (4.8×10^−8^)	3.17×10^−6^ (5.1×10^−6^)	5.72×10^−7^ (1.3×10^−6^)	2.55×10^−8^ (3.3×10^−8^)
**MRSA**						
FOF	2.86×10^−5^ (3.6×10^−5^)	2.07×10^−5^ (3.1×10^−5^)	2.78×10^−5^ (3.2×10^−5^)	1.73×10^−6^ (3.9×10^−6^)	4.57×10^−8^ (2.3×10^−8^)	3.57×10^−8^ (8.2×10^−9^)
TOB	1.2×10^−5^ (3.7×10^−6^)	3.87×10^−6^ (1.7×10^−6^)	5.59×10^−7^ (5.7×10^−7^)	3.13×10^−5^ (4.5×10^−5^)	2.92×10^−6^ (2.9×10^−6^)	1.04×10^−6^ (1.3×10^−6^)
F∶T	5.05×10^−7^ (6.9×10^−7^)	2.76×10^−7^ (6×10^−7^)	2.88×10^−8^ (7.8×10^−9^)	3.57×10^−8^ (8.2×10^−9^)	3.57×10^−8^ (8.2×10^−9^)	3.57×10^−8^ (8.2×10^−9^)

a Where the spontaneous mutation frequency was above or below the limit of detection, the limit of detection was used to calculate the mean e.g. if spontaneous mutation frequency was <4.4×10^−8^ then 4.4×10^−8^ was used.

MRSA isolates (n = 6) also had a lower frequency of spontaneous mutants following exposure to F∶T compared to fosfomycin and tobramycin under both aerobic and anaerobic conditions (Table F∶T 2 and Table F∶T S3, for full results). The frequency of mutants on exposure to F∶T was below the detectable limit in 32/36 (89%) experiments. In contrast, the frequency of MRSA mutants to fosfomycin and tobramycin was below the limit of detection in only 15/36 (42%) and 6/36 (17%) experiments, respectively. MRSA isolates had lower mean frequency of mutants to fosfomycin under anaerobic (average 3.57×10^−8^ at 8× MIC) as compared to aerobic conditions (average 2.78×10^−5^ at 8× MIC) whereas the frequency of mutants to tobramycin MICs was higher under anaerobic (average 1.04×10^−6^ at 8× MIC) compared to aerobic (average 5.59×10^−7^ at 8× MIC) conditions.

### Spontaneous *P. aeruginosa* and MRSA mutants resistant to fosfomycin or tobramycin alone did not have elevated F∶T MICs

Spontaneous mutant isolates which developed resistance following exposure to fosfomycin or tobramycin did not show any increase in F∶T MIC under either aerobic or anaerobic conditions indicating that isolates resistant to either agent alone were not cross resistant to the combination (see Table S4).

### F∶T MICs did not increase when *P. aeruginosa* and MRSA isolates were passaged in sub-inhibitory concentrations of F∶T

**Figure 1:**
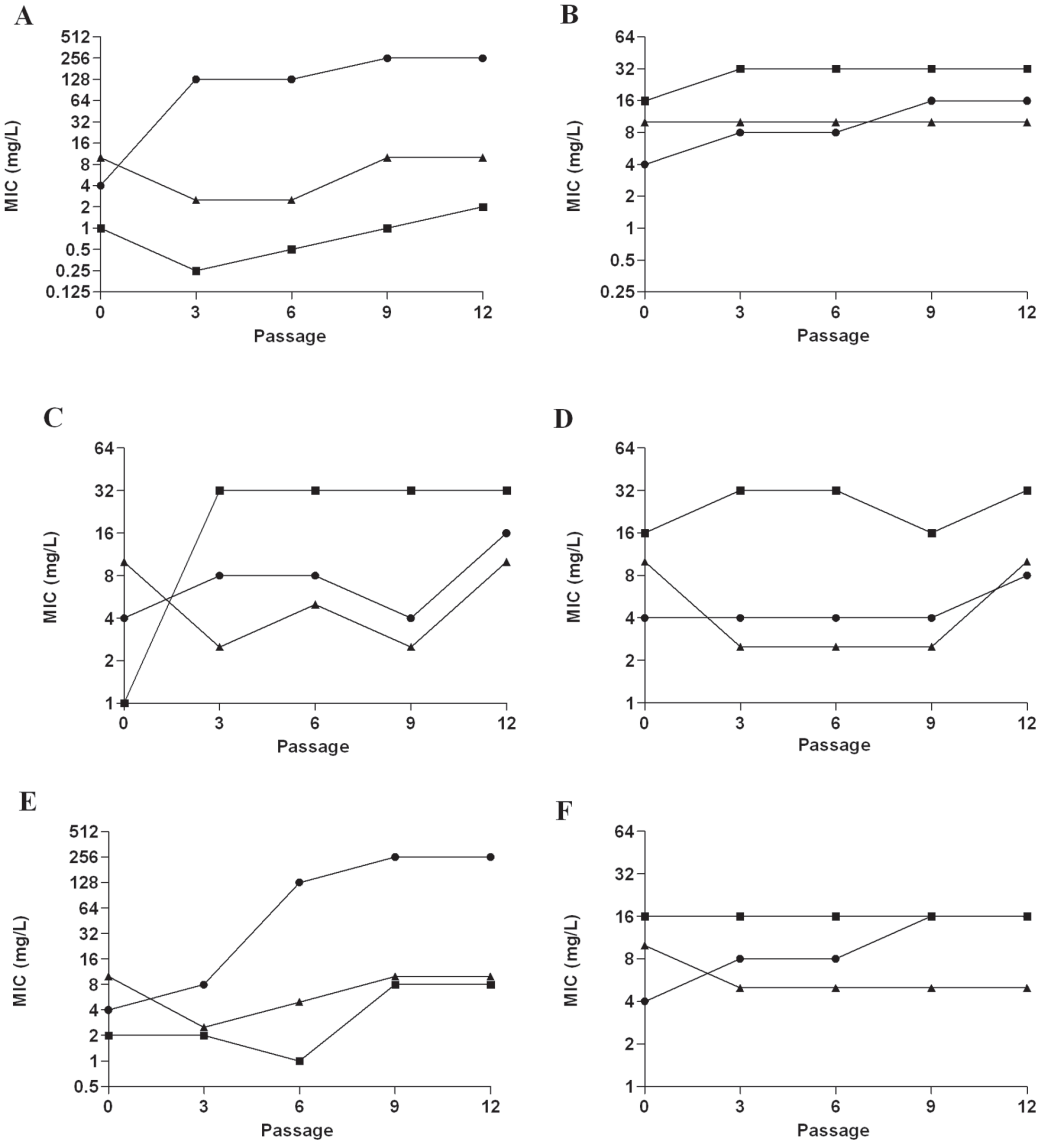
Fosfomycin, tobramycin and F∶T MICs for *P. aeruginosa* isolate W050 following serial passage in sub-inhibitory antibiotic concentrations under both aerobic and anaerobic conditions. Exposure to (A) Fosfomycin aerobic, (B) Fosfomycin anaerobic, (C) Tobramycin aerobic, (D) Tobramycin anaerobic, (E) F∶T aerobic, (F) F∶T anaerobic. Filled circle (•) Fosfomycin MIC; filled square (▪) Tobramycin MIC; filled triangle (▴)F∶T MIC.

**Figure 2:**
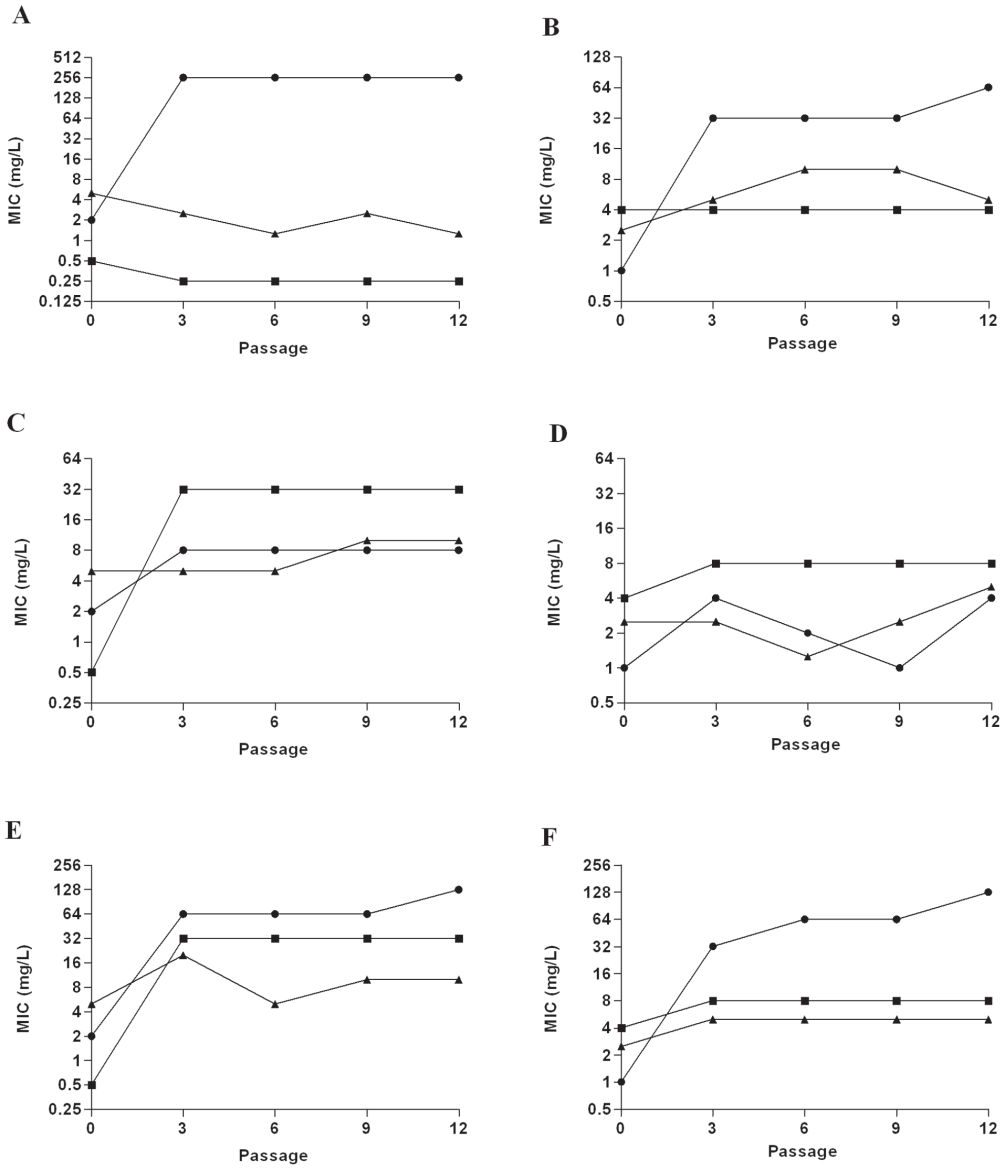
Fosfomycin, tobramycin and F∶T MICs for MRSA isolate CFP8 following serial passage in sub-inhibitory antibiotic concentrations under both aerobic and anaerobic conditions. Exposure to (A) Fosfomycin aerobic, (B) Fosfomycin anaerobic, (C) Tobramycin aerobic, (D) Tobramycin anaerobic, (E) F∶T aerobic, (F) F∶T anaerobic. Filled circle (•) Fosfomycin MIC; filled square (▪) Tobramycin MIC; filled triangle (▴)F∶T MIC.

*P. aeruginosa* (n = 3) and MRSA (n = 3) isolates were passaged in sub-inhibitory concentrations of fosfomycin, tobramycin and F∶T to determine if resistance could be induced by antibiotic exposure. Results for representative *P. aeruginosa* and MRSA isolates shown in figures 1 and 2, respectively, with all results shown in Table S5. *P. aeruginosa* isolates exhibited no increase in F∶T MIC after passage in sub-inhibitory F∶T under both aerobic and anaerobic conditions; however, there was a 4-fold increase in the tobramycin MIC of one of the isolates when grown in sub-inhibitory F∶T under aerobic conditions. When grown in sub-inhibitory fosfomycin, 2/3 and 1/3 *P. aeruginosa* isolates developed resistance under aerobic and anaerobic conditions, respectively. When grown in sub-inhibitory tobramycin, 1/3 *P. aeruginosa* isolates developed resistance under aerobic conditions. Under anaerobic conditions, all three *P. aeruginosa* isolates were classified as resistant to tobramycin prior to antibiotic exposure and remained resistant to tobramycin during passage in sub-inhibitory antibiotics.

Following passage in sub-inhibitory F∶T, 1/3 and 3/3 MRSA isolates demonstrated a 2-fold increase in F∶T MIC under aerobic and anaerobic conditions, respectively. Fosfomycin MIC increases of 4–64 fold and 64–128 fold were also observed under aerobic and anaerobic conditions, respectively; however, none of the isolates post-exposure were categorised as resistant to fosfomycin. All 3 MRSA isolates developed resistance to fosfomycin when grown in sub-inhibitory fosfomycin under aerobic conditions and although MICs did increase from 32- to 64-fold under anaerobic conditions, isolates were not categorised as resistant to fosfomycin. When grown in sub-inhibitory tobramycin, 1/3 and 2/3 MRSA isolates developed resistance under aerobic and anaerobic conditions, respectively.

### Induced tobramycin and fosfomycin resistance is stable after removal of antibiotic pressure and not associated with an *in vitro* biological fitness cost

**Figure 3:**
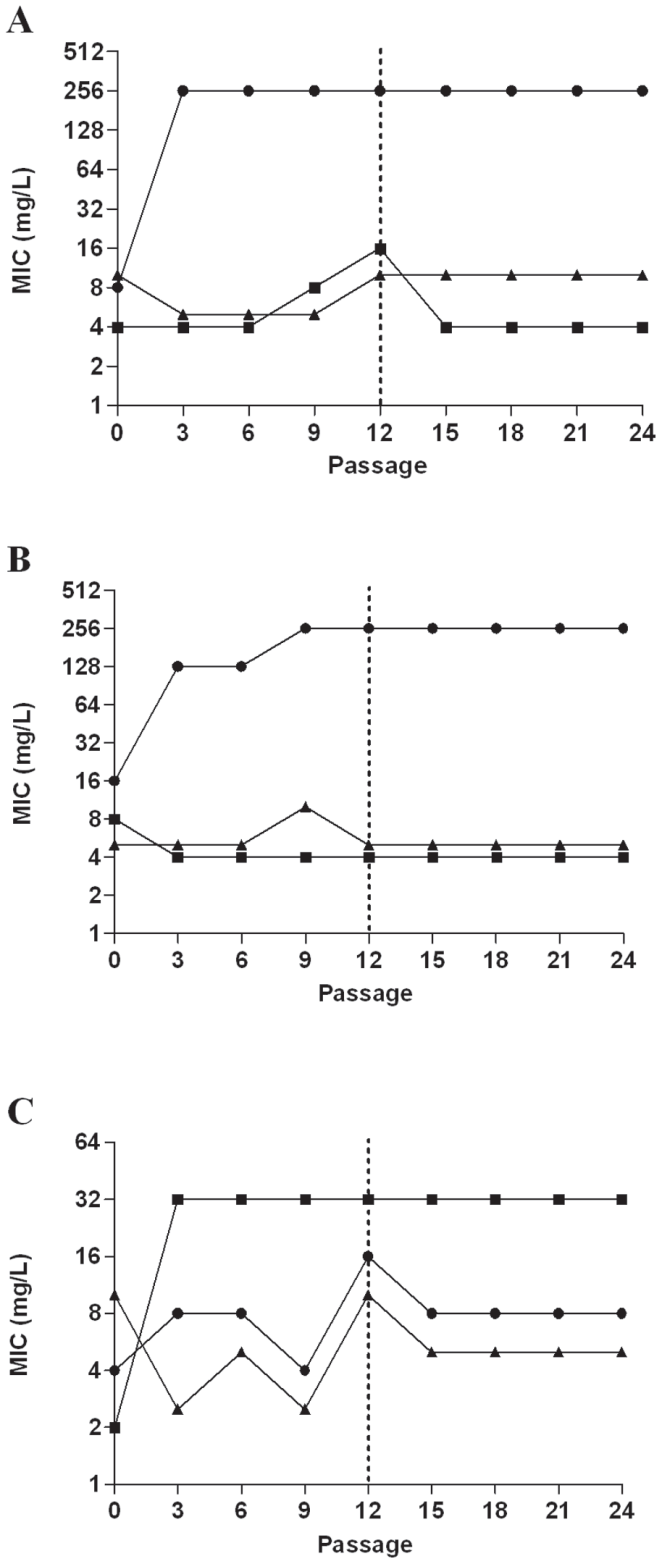
Fosfomycin, tobramycin and F∶T MICs for *P. aeruginosa* isolates following serial passage in sub-inhibitory antibiotic concentrations and subsequent removal of antibiotic pressure following 12 passages. (A) AY4 FOS aerobic, (B) AY4 FOS anaerobic and (C) W050 TOB aerobic. Filled circle (•) Fosfomycin MIC; filled square (▪) Tobramycin MIC; filled triangle (▴)F∶T MIC; broken line, antibiotic pressure removed.

**Figure 4:**
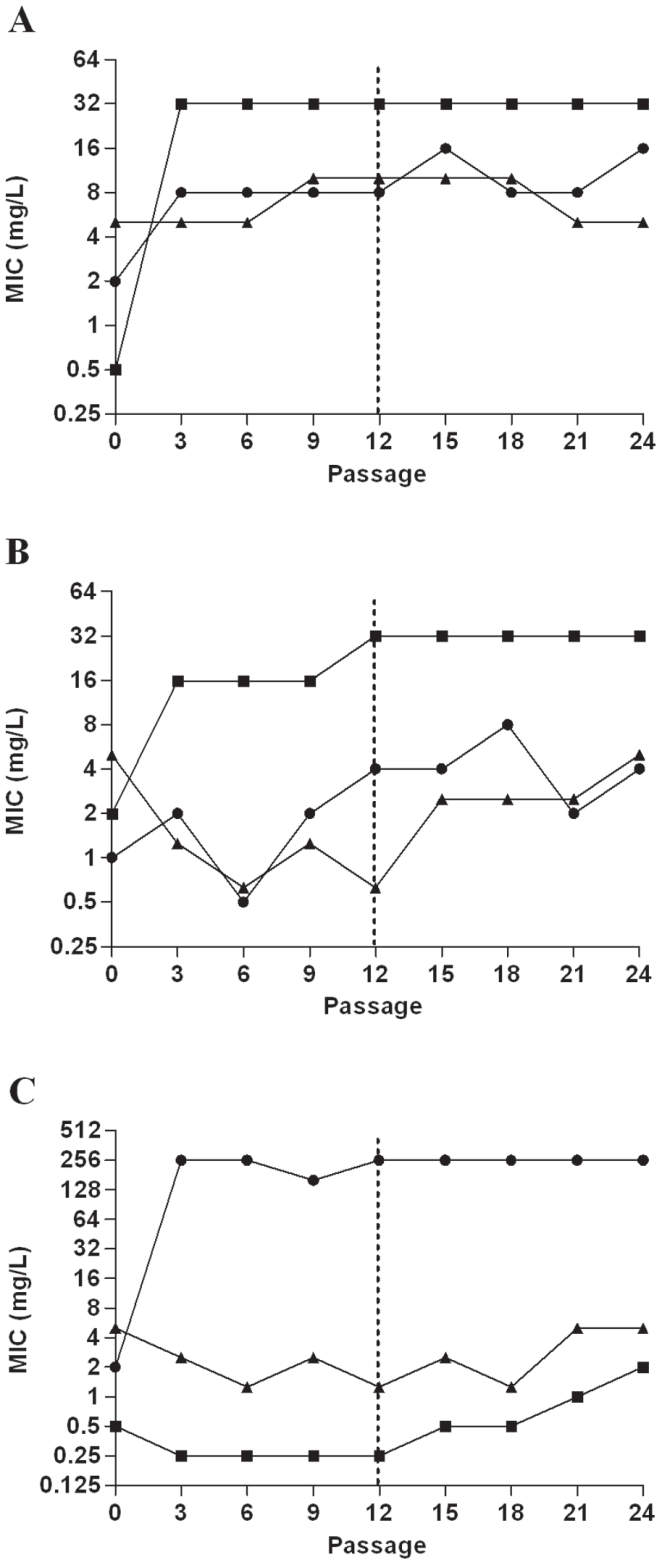
Fosfomycin, tobramycin and F∶T MICs for MRSA isolates following serial passage in sub-inhibitory antibiotic concentrations and subsequent removal of antibiotic pressure following 12 passages. (A) CFP8 TOB aerobic, (B) 25A TOB anaerobic, (C) CFP8 FOF aerobic. Filled circle (•) Fosfomycin MIC; filled square (▪) Tobramycin MIC; filled triangle (▴),F∶T MIC, broken line, antibiotic pressure removed.

**Figure 5:**
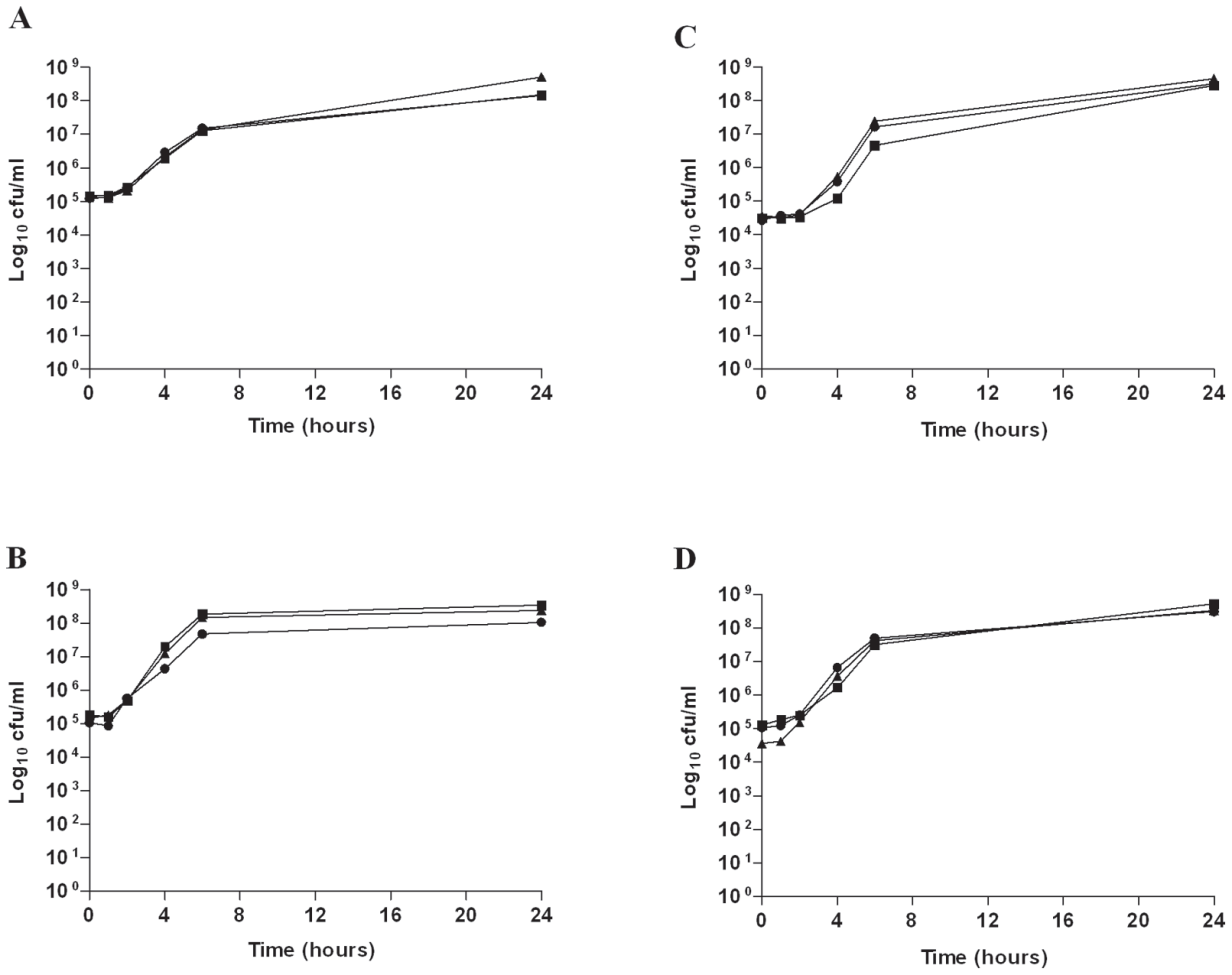
Growth curves showing total viable counts of parent MRSA and *P. aeruginosa* isolates and resistant mutants which developed following exposure to fosfomycin and tobramycin. (A) MRSA CFP13, aerobic, (B) MRSA CFP13 anaerobic, (C) *P. aeruginosa* W050 aerobic and (D) *P. aeruginosa* W050 anaerobic. Filled circle (•) Parent strain; filled square (▪) Fosfomycin resistant mutant; filled triangle (▴) Tobramycin resistant mutant.

*P. aeruginosa* and MRSA isolates which developed resistance to fosfomycin (n = 7; 3 *P. aeruginosa*, 4 MRSA) or tobramycin (n = 6; 3 *P. aeruginosa*, 3 MRSA) after serial exposure to sub-inhibitory fosfomycin or tobramycin remained resistant after 12 passages in drug free broth. Results for representative *P. aeruginosa* and MRSA isolates are shown in Figures 3 and 4, respectively. There was no fitness cost associated with acquisition of fosfomycin or tobramycin resistance in *P. aeruginosa* or MRSA isolates under either aerobic or anaerobic conditions; this was demonstrated by no differences in viable counts between the resistant isolates and the parent strain over a 24 hour period, as shown in Figure 5 for representative isolates.

## Discussion

Due to the widespread use of antibiotics in the prophylaxis and treatment of CF pulmonary infection, pathogens such as *P. aeruginosa* and MRSA are becoming increasingly resistant to conventional anti-pseudomonal and anti-staphylococcal agents. Antibiotics are frequently used in combination in CF with the aim of providing better coverage of target organisms and preventing or slowing the development of antimicrobial resistance [16]; however there is limited data on the ability of such combinations to prevent resistance development in this setting. In the present study, we have shown that the combination of fosfomycin and tobramycin in F∶T can prevent the onset of resistance compared to either agent alone in clinical CF *P. aeruginosa* and MRSA isolates. This study is also the first to investigate the effect of anaerobic conditions on the development of resistance by CF pathogens with resistance development to both fosfomycin and F∶T less likely under anaerobic conditions.

Both *P. aeruginosa* and MRSA had a lower frequency of spontaneous mutants to F∶T compared to fosfomycin or tobramycin alone, with spontaneous mutants to F∶T also less likely to arise under physiologically relevant anaerobic conditions. The low spontaneous mutation frequencies observed with F∶T indicate that the development of resistance by spontaneous mutation to this combination is unlikely. Two previous studies also showed that *P. aeruginosa* and *S. aureus* had a low mutation frequency to a combination of fosfomycin and tobramycin under aerobic conditions, making the emergence of resistance unlikely [15], [17]. Similarly, a decrease in both mutation frequency and development of resistance has also been reported for *P. aeruginosa* and MRSA when fosfomycin was combined with other antibiotics such as linezolid, perfloxacin, ciprofloxacin and amikacin [17]–[19].

In contrast to F∶T, both *P. aeruginosa* and MRSA isolates had high mutation frequencies to fosfomycin and tobramycin alone under both aerobic and anaerobic conditions. These results are consistent with previous studies which have shown that Gram negative pathogens such as *Escherichia coli* have high mutation frequencies against fosfomycin [20], [21]. Similarly, MacLeod *et al*. also demonstrated that both MRSA and *P. aeruginosa* had high mutation frequencies against both fosfomycin and tobramycin under aerobic conditions [15]. The high mutation frequencies of these pathogens to fosfomycin and tobramycin indicate that resistance development would be extremely likely if these agents were used as monotherapy to treat infections caused by *P. aeruginosa* or MRSA, potentially leading to treatment failure.

In the CF lung, bacteria are often subjected to sub-lethal concentrations of antibiotics, as the ionic CF lung environment reduces drug uptake and often prevents antibiotics reaching bactericidal levels [22], [23]. Induction of resistance experiments showed that there were no more than 2-fold increases in F∶T MICs when MRSA and *P. aeruginosa* isolates were grown in sub-inhibitory F∶T over 12 passages under both aerobic or anaerobic conditions. Our results are in agreement with those which previously showed that combining tobramycin with other antibacterial agents can delay or prevent the onset of resistance compared to either single agent alone [24]. In contrast to F∶T, resistance to fosfomycin or tobramycin developed quickly in both *P. aeruginosa* and MRSA isolates under both conditions. As fosfomycin is not routinely used in the treatment of CF lung infections, little data on fosfomycin resistance patterns amongst CF isolates is available. However, there has been a significant increase in the prevalence of aminoglycoside resistance in *P. aeruginosa* isolated from CF patients in recent years [5]. Both intravenous [25] and inhaled [26], [27] tobramycin therapy has been associated with a trend towards higher MICs and resistance amongst *P. aeruginosa* isolates. In addition, long term tobramycin therapy has been shown to be a risk factor for the development of multi-drug resistant *P. aeruginosa* in CF [28]. The increased prevalence of aminoglycoside resistance in CF and the speed at which resistance to tobramycin developed in the current study may be due to the fact that sub-lethal concentrations of aminoglycosides, such as tobramycin, induce mutagenesis in *P. aeruginosa in vitro* [22]. Our results suggest that if tobramycin or fosfomycin were to be used alone, sub-lethal concentrations in sputum would drive resistance, thus highlighting the importance of using the combination of fosfomycin and tobramycin in F∶T to prevent the development of resistance. In agreement with our findings, Trapnell et al. have recently shown, in a clinical trial, that when Fosfomycin: Tobramycin for Inhalation (FTI) was used with *P. aeruginosa* positive CF patients, there was no increase in fosfomycin, tobramycin or FTI MICs (MIC_50_ and MIC_90_) following 28 days treatment [29].

While the results of the current study have shown that resistance to both fosfomycin and tobramycin developed quickly *in vitro*, it is unclear whether there is always a correlation between *in vitro* and *in vivo* resistance development. A recent review [30] concluded that there is a lack of correlation between *in vitro* development of fosfomycin resistance in *E. coli* and *in vivo* resistance development; however, the review also concluded that development of resistance was more likely for *P. aeruginosa*. Lack of correlation between *in vitro* and *in vivo* resistance has traditionally been attributed to a biological fitness cost associated with the acquisition of resistance [31]. Fitness cost is an important determinant of the ability of the mutant bacteria to survive and propagate in a given biological niche, with the frequency of a resistant mutant in a bacterial population being inversely proportional to the fitness cost it carries [32]. Our results indicate that there is no fitness cost associated with the development of fosfomycin or tobramycin resistance in *P. aeruginosa* or MRSA isolates under either aerobic or anaerobic conditions. This suggests that isolates resistant to fosfomycin or tobramycin would have the ability to survive and propagate in the CF lung *in vivo*, potentially leading to treatment failure if these agents were used as monotherapy. Similarly, it has been reported that there is no fitness cost associated with fosfomycin resistance in *P. aeruginosa* isolates under aerobic conditions in an *in vivo* acute mice lung infection model [33]. Furthermore, our results indicate that acquired fosfomycin or tobramycin resistance is stable in these pathogens and remains even after the removal of antibiotic pressure. This suggests that these resistant isolates would persist in the CF lung and would continue to be recalcitrant to fosfomycin or tobramycin therapy.

Isolates which developed resistance to either fosfomycin or tobramycin alone did not have elevated F∶T MICs indicating that the combination remained effective against isolates resistant to either antibiotic alone. This finding and the fact that resistance to F∶T did not develop may be due to the different modes of action of fosfomycin and tobramycin. Fosfomycin inhibits an early step in peptidoglycan synthesis in the bacterial cell wall whereas tobramycin primarily exerts its effect on the bacterial ribosome. It is generally accepted that bacteria are less likely to develop resistance to two agents with different modes of action as this will require two separate mutational events, conferring resistance to each antibiotic. It has also recently been shown that, under aerobic conditions, fosfomycin enhances the uptake of tobramycin resulting in increased inhibition of protein synthesis and bacterial killing [34]. This interaction between the two antibiotics may also explain why isolates resistant to either agent are not resistant to the combination; for example, mutations conferring resistance to fosfomycin may not prevent it acting in synergy to increase intracellular levels of tobramycin.

In conclusion, the results of this study suggest that F∶T may prevent the development of resistance compared to fosfomycin or tobramycin alone under aerobic and physiologically relevant anaerobic conditions. As such, F∶T may be a promising treatment for CF patients colonised by *P. aeruginosa* or MRSA isolates, particularly for use as a chronic maintenance therapy due to the low likelihood of resistance development.

## Supporting Information

Table S1 Concentrations of fosfomycin (FOF), tobramycin (TOB) and F: T used in induction of resistance studies.
(DOCX)

Table S2 Frequency of spontaneous *P. aeruginosa* mutants with increased fosfomycin (FOF), tobramycin (TOB) and F∶T MICs under aerobic and anaerobic conditions.
(DOCX)

Table S3 Frequency of spontaneous MRSA mutants with increased fosfomycin (FOF), tobramycin (TOB) and F∶T MICs under aerobic and anaerobic conditions.
(DOCX)

Table S4 Fosfomycin, tobramycin and F∶T MICs of selected spontaneous mutants under aerobic and anaerobic conditions.
(DOCX)

Table S5 Fosfomycin, tobramycin and F∶T MICs of *P. aeruginosa* and MRSA isolates when passaged in sub-inhibitory fosfomycin, tobramycin and F∶T for 12 passages in induction of resistance experiments.
(XLSX)
