# Supplementary material for: Resistance Development of Cystic Fibrosis Respiratory Pathogens When Exposed to Fosfomycin and Tobramycin Alone and in Combination under Aerobic and Anaerobic Conditions
Source: PLoS One. 2013 Jul 25;8(7):e69763. doi: 10.1371/journal.pone.0069763 (PMC3723830; doi:10.1371/journal.pone.0069763)
Supplement: Table S1 — Concentrations of fosfomycin (FOF), tobramycin (TOB) and F: T used in induction of resistance studies. (DOCX) [file pone.0069763.s001.docx]

Table S1. Concentrations of fosfomycin (Fof), tobramycin (Tob) and F:T used in induction of resistance studies

| Species | Aerobic (mg/L) | | | Anaerobic (mg/L) | | | |
| --- | --- | --- | --- | --- | --- | --- | --- |
|  | Fof | Tob | F:T | | Fof | Tob | F:T |
| *P. aeruginosa* | 1 | 0.25 | 1.25 | | 1 | 0.25 | 1.25 |
|  |  |  |  | |  |  |  |
| MRSA | 0.5 | 0.125 | 0.625 | | 0.5 | 0.125 | 0.625 |
