# Supplementary material for: Resistance Development of Cystic Fibrosis Respiratory Pathogens When Exposed to Fosfomycin and Tobramycin Alone and in Combination under Aerobic and Anaerobic Conditions
Source: PLoS One. 2013 Jul 25;8(7):e69763. doi: 10.1371/journal.pone.0069763 (PMC3723830; doi:10.1371/journal.pone.0069763)
Supplement: Table S2 — Frequency of spontaneous P. aeruginosa mutants with increased fosfomycin (FOF), tobramycin (TOB) and F∶T MICs under aerobic and anaerobic conditions. (DOCX) [file pone.0069763.s002.docx]

Table S2. Frequency of spontaneous *P. aeruginosa* mutants with increased fosfomycin (FOF), tobramycin (TOB) and F:T MICs under aerobic and anaerobic conditions at 2x, 4x and 8x MIC.

| **Isolate** | **Selecting Drug** | **Aerobic** | | | **Anaerobic** | | |
| --- | --- | --- | --- | --- | --- | --- | --- |
|  |  | **2 x MIC** | **4 x MIC** | **8 X MIC** | **2 x MIC** | **4 x MIC** | **8 X MIC** |
| CF35 | FOF | 1.82 x 10^-5^ | 9.79 x 10^-6^ | 5.28 x 10^-7^ | 3.31 x 10^-6^ | 1.52 x 10^-6^ | 2.75 x 10^-7^ |
|  | TOB | 1.07 x 10^-6^ | 3.56 x 10^-6^ | <2.72 x 10^-8^ | >2.29 x 10^-4^ | 6.80 x 10^-6^ | 2.34 x 10^-6^ |
|  | F:T | <2.72 x 10^-8^ | <2.72 x 10^-8^ | <2.72 x 10^-8^ | <4.59 x 10^-8^ | <4.59 x 10^-8^ | <4.59 x 10^-8^ |
| P3 | FOF | 1.15 x 10^-6^ | 1.19 x 10^-6^ | 6.06 x 10^-6^ | 6.12 x 10^-6^ | 2.89 x 10^-6^ | <8.69 x 10^-9^ |
|  | TOB | > 6.06 x 10^-6^ | >6.06 x 10^-6^ | 8.48 x 10^-9^ | 1.32 x 10^-5^ | 6.02 x 10^-6^ | <8.69 x 10^-9^ |
|  | F:T | 1.20 x 10^-7^ | <1.21 x 10^-9^ | <1.21 x 10^-9^ | <8.69 x 10^-9^ | <8.69 x 10^-9^ | <8.69 x 10^-9^ |
| W050 | FOF | >1.2 x 10^-4^ | >1.2 x 10^-4^ | >1.2 x 10^-4^ | >2.99 x 10^-5^ | 7.86 x 10^-6^ | 9.79x 10^-7^ |
|  | TOB | <2.41 x 10^-8^ | <2.41 x 10^-8^ | <2.41 x 10^-8^ | <5.97x 10^-9^ | <5.97x 10^-9^ | <5.97x 10^-9^ |
|  | F:T | <2.41 x 10^-8^ | <2.41 x 10^-8^ | <2.41 x 10^-8^ | <5.97x 10^-9^ | <5.97x 10^-9^ | <5.97x 10^-9^ |
| CA6 | FOF | 3.27 x 10^-7^ | 2.33 x 10^-7^ | <6.67 x 10^-9^ | 4.66 x 10^-6^ | 7.32 x 10^-7^ | <8.51 x 10^-9^ |
|  | TOB | >3.33 x 10^-5^ | 3.72 x 10^-5^ | 2.20 x 10^-5^ | >4.25 x 10^-6^ | >4.25 x 10^-6^ | >4.25 x 10^-5^ |
|  | F:T | <6.67 x 10^-9^ | <6.67 x 10^-9^ | <6.67 x 10^-9^ | 8.51 x 10^-8^ | <8.51 x 10^-9^ | <8.51 x 10^-9^ |
| AY4 | FOF | 3.77 x 10^-5^ | 2.4 x 10^-5^ | 7.11 x 10^-6^ | 3.59 x 10^-6^ | 2.14 x 10^-6^ | 3.62 x 10^-7^ |
|  | TOB | <1.11 x 10^-7^ | <1.11 x 10^-7^ | <1.11 x 10^-7^ | >1.18 x 10^-5^ | >1.18 x 10^-5^ | >1.18 x 10^-5^ |
|  | F:T | <1.11 x 10^-7^ | <1.11 x 10^-7^ | <1.11 x 10^-7^ | >1.18 x 10^-5^ | 3.28 x 10^-6^ | <2.35x 10^-9^ |
| 27853 | FOF | >2.2 x 10 ^-4^ | 1.19 x 10^-5^ | 8.36 x 10^-7^ | >2.6 x 10^-5^ | >2.6 x 10^-5^ | >2.6 x 10^-5^ |
|  | TOB | <4.40 x 10^-8^ | <4.40 x 10^-8^ | <4.40 x 10^-8^ | 1.65 x 10^-6^ | 1.19x 10^-7^ | <5.19 x 10^-9^ |
|  | F:T | <4.40 x 10^-8^ | <4.40 x 10^-8^ | <4.40 x 10^-8^ | 7.1x 10^-6^ | <5.19 x 10^-9^ | <5.19 x 10^-9^ |
