# Supplementary material for: Resistance Development of Cystic Fibrosis Respiratory Pathogens When Exposed to Fosfomycin and Tobramycin Alone and in Combination under Aerobic and Anaerobic Conditions
Source: PLoS One. 2013 Jul 25;8(7):e69763. doi: 10.1371/journal.pone.0069763 (PMC3723830; doi:10.1371/journal.pone.0069763)
Supplement: Table S3 — Frequency of spontaneous MRSA mutants with increased fosfomycin (FOF), tobramycin (TOB) and F∶T MICs under aerobic and anaerobic conditions. (DOCX) [file pone.0069763.s003.docx]

Table S3. Frequency of spontaneous MRSA mutants with increased fosfomycin (FOF), tobramycin (TOB) and F:T MICs under aerobic and anaerobic conditions at 2x, 4x and 8x MIC.

| **Isolate** | **Selecting Drug** | **Aerobic** | | | **Anaerobic** | | |
| --- | --- | --- | --- | --- | --- | --- | --- |
|  |  | **2 x MIC** | **4 x MIC** | **8 X MIC** | **2 x MIC** | **4 x MIC** | **8 X MIC** |
| 25A | FOF | 6.3 x 10^-6^ | 4.7 x 10^-7^ | 5.6 x 10^-7^ | 9.6 x 10^-6^ | <3.8 x 10^-8^ | <3.8 x 10^-8^ |
|  | TOB | 1.7 x 10^-5^ | 7 x 10^-6^ | 1.5 x 10^-6^ | <3.8 x 10^-8^ | <3.8 x 10^-8^ | <3.8 x 10^-8^ |
|  | F:T | 5.6 x 10^-7^ | <2.5 x 10^-8^ | <2.5 x 10^-8^ | <3.8 x 10^-8^ | <3.8 x 10^-8^ | <3.8 x 10^-8^ |
| CFP13 | FOF | 7.5 x 10^-5^ | 6.4 x 10^-5^ | 6.3 x 10^-5^ | 4.5 x 10^-7^ | <3 x 10^-8^ | <3 x 10^-8^ |
|  | TOB | 1.4 x 10^-5^ | 2.8 x 10^-6^ | 2.3 x 10^-8^ | 1.2 x 10^-4^ | 4.5 x 10^-6^ | 1.9 x 10^-6^ |
|  | F:T | <2.3 x 10^-8^ | <2.3 x 10^-8^ | <2.3 x 10^-8^ | <3 x 10^-8^ | <3 x 10^-8^ | <3 x 10^-8^ |
| M6 | FOF | 3.6 x 10 ^-6^ | 5.1 x 10^-7^ | 6.8 x 10^-5^ | <4 x 10^-8^ | <4 x 10^-8^ | <4 x 10^-8^ |
|  | TOB | 6.4 x 10^-6^ | 2.5 x 10^-6^ | 1.7 x 10^-7^ | 2.2 x 10^-5^ | 6.5 x 10^-6^ | 9.2 x 10^-7^ |
|  | F:T | <3.4 x 10^-8^ | <3.4 x 10^-8^ | <3.4 x 10^-8^ | <4 x 10^-8^ | <4 x 10^-8^ | <4 x 10^-8^ |
| M10 | FOF | 2.6 x 10^-6^ | 4.1 x 10^-7^ | <4.1 x 10^-8^ | <4.9 x 10^-8^ | <4.9 x 10^-8^ | <4.9 x 10^-8^ |
|  | TOB | 1.2 x 10^-5^ | 2.6 x 10^-6^ | 2.5 x 10^-7^ | 3.2 x 10^-5^ | 8.3 x 10^-7^ | <4.9 x 10^-8^ |
|  | F:T | 5.8 x 10^-7^ | <4.1 x 10^-8^ | <4.1 x 10^-8^ | <4.9 x 10^-8^ | <4.9 x 10^-8^ | <4.9 x 10^-8^ |
| CFP8 | FOF | 7.6 x 10^-5^ | 5.8 x 10^-5^ | 3.5 x 10^-5^ | <2.8 x 10^-8^ | <2.8 x 10^-8^ | <2.8 x 10^-8^ |
|  | TOB | 9.3 x 10^-6^ | 4.3 x 10^-6^ | 9.7 x 10^-7^ | 1.3 x 10^-5^ | 5.6 x 10^-6^ | 3.3 x 10^-6^ |
|  | F:T | 1.8 x 10^-6^ | 1.5 x 10^-6^ | <2 x 10^-8^ | <2.8 x 10^-8^ | <2.8 x 10^-8^ | <2.8 x 10^-8^ |
| 29213 | FOF | 8.1 x 10^-6^ | 9.9 x 10^-7^ | 3 x 10^-7^ | 2.1 x 10^-7^ | 8.9 x 10^-8^ | <2.9 x 10^-8^ |
|  | TOB | 1.3 x 10^-5^ | 4 x 10^-6^ | 4.4 x 10^-7^ | 6.5 x 10^-7^ | <2.9 x 10^-8^ | <2.9 x 10^-8^ |
|  | F:T | <3 x 10^-8^ | <3 x 10^-8^ | <3 x 10^-8^ | <2.9 x 10^-8^ | <2.9 x 10^-8^ | <2.9 x 10^-8^ |
