# Supplementary material for: Resistance Development of Cystic Fibrosis Respiratory Pathogens When Exposed to Fosfomycin and Tobramycin Alone and in Combination under Aerobic and Anaerobic Conditions
Source: PLoS One. 2013 Jul 25;8(7):e69763. doi: 10.1371/journal.pone.0069763 (PMC3723830; doi:10.1371/journal.pone.0069763)
Supplement: Table S4 — Fosfomycin, tobramycin and F∶T MICs of selected spontaneous mutants under aerobic and anaerobic conditions. (DOCX) [file pone.0069763.s004.docx]

Table S4. Fosfomycin, tobramycin and F:T MICs for selected *P. aeruginosa* (27853, AY4) and MRSA (CFP8) strains under control conditions and corresponding spontaneous mutants after selection with Fosfomycin or tobramycin under aerobic and anaerobic conditions.

| Strain/ Growth condition | MIC (mg/L) control | | | Spontaneous mutant selected with | MIC (mg/L) post selection | | |
| --- | --- | --- | --- | --- | --- | --- | --- |
|  | **Fos** | **Tob** | **F:T** |  | **Fos** | **Tob** | **F:T** |
| AY4/ Aerobic | 8 | 4 | 10 | Fosfomcyin | 256 | 4 | 10 |
| 27853/ Aerobic | 4 | 1 | 5 | Fosfomcyin | 256 | 1 | 5 |
| CFP8/Aerobic | 2 | 0.5 | 5 | Fosfomcyin | 256 | 0.5 | 5 |
| AY4/ Anaerobic | 16 | 8 | 10 | Fosfomcyin | 256 | 16 | 10 |
| CFP8/ Anaerobic | 1 | 4 | 2.5 | Fosfomcyin | 128 | 4 | 2.5 |
|  |  |  |  |  |  |  |  |
| 27853/ Aerobic | 4 | 1 | 5 | Tobramycin | 2 | 8 | 5 |
| CFP8/ Aerobic | 2 | 0.5 | 5 | Tobramycin | 4 | 32 | 5 |
| AY4/ Anaerobic | 16 | 8 | 5 | Tobramycin | 8 | 32 | 2.5 |
| CFP8/ Anaerobic | 1 | 4 | 2.5 | Tobramycin | 1 | 32 | 2.5 |

^a^ S = susceptible, I = intermediate, R = resistant according to CLSI breakpoints
